# Changes in Skeletal Muscle Mass in the First 3 Months Following Gastrointestinal Cancer Surgery: A Prospective Study

**DOI:** 10.1245/s10434-024-16109-8

**Published:** 2024-09-04

**Authors:** Xinyi Xu, Wei-Hong Liu, Patsy Yates

**Affiliations:** Cancer and Palliative Care Outcomes Centre, Centre for Healthcare Transformation, School of Nursing, Faculty of Health, Queensland University of Technology, Brisbane, Australia

**Keywords:** Gastrointestinal cancer, Growth mixture modeling, Predictor, Quality of life, Sarcopenia, Skeletal muscle

## Abstract

**Background:**

Surgical resection is the primary treatment for gastrointestinal (GI) cancers, but postoperative skeletal muscle loss (SML) is common and linked to poor prognosis. This study aims to identify patterns of muscle change, examine its association with quality of life (QoL), and explore predictors of SML in the first 3 months.

**Patients and Methods:**

A prospective cohort study was conducted on patients newly diagnosed with GI cancer and undergoing surgery in China between September 2021 and May 2022. Skeletal muscle mass (SMM) and QoL were assessed at admission, 7 days, 1 month, and 3 months post-surgery. Demographic, clinical data, and biomarkers were collected. Missing data were imputed using multiple imputation. Data were analyzed using growth mixture modelling, bivariate analyses, and logistic regression.

**Results:**

A total of 483 patients completed baseline assessment. Of the 242 patients with complete muscle assessments, 92% experienced SML. Three distinct patterns of muscle change were identified: 57% had normal preoperative SMM with mild postoperative SML, 16% had low preoperative SMM with moderate SML, and 27% had normal preoperative mass but severe postoperative SML. Moderate/severe SML was associated with more postoperative complications, poorer health, and higher symptom burden. Independent predictors included advanced age, preoperative sarcopenia, advanced cancer stage, and low prognostic nutrition index (PNI ≤ 45). The results did not change when using imputed values.

**Conclusions:**

Although SML is prevalent, patterns of muscle change are heterogeneous among patients. Advanced age, preoperative sarcopenia, advanced cancer stage, and cancer-related inflammation are predictors for moderate/severe SML, highlighting the need for early detection and management.

**Supplementary Information:**

The online version contains supplementary material available at 10.1245/s10434-024-16109-8.

Colorectal, gastric, and esophageal cancers are common gastrointestinal (GI) cancers that account for 19% of the total new cancer cases and 23% of the total cancer-related deaths worldwide each year.^1^ China has the highest number of patients with GI cancer in the world, accounting for nearly 25% of the global colorectal cancer cases and 50% of global gastric and esophageal cancer cases.^2^ Surgical resection is recommended as the primary curative treatment for localized GI cancers.^3^ However, major surgical procedures alter the anatomical structure and function of the GI tract. Consequently, patients often experience nausea, diarrhea, anorexia, and dumping syndrome after surgery,^4^ which can result in the malabsorption, micronutrient deficiencies, and unintentional weight loss.^5,6^ Postoperative weight loss has been reported to be associated with a range of adverse health outcomes and increased risk of recurrence and mortality in patients with GI cancer.^7,8^

A consensus is emerging that body weight may not be a concise prognostic indicator for clinical outcomes in patients with cancer. Patients with similar body weight can exhibit different distribution of skeletal muscle mass (SMM) and fat mass.^9^ In addition, the presence of edema and ascites in patients with GI cancer can affect the reliability of body weight measurements.^10^ Hence, there is an increased interest in using SMM as a prognosis indicator.^11–14^ Indeed, some studies have shown that skeletal muscle loss (SML) is associated with poorer long-term survival outcomes in GI cancer.^12–14^ However, most of these studies were retrospective and yet included patient-reported health outcomes, such as cancer and treatment-related symptoms and quality of life (QoL). Additionally, these studies varied greatly in terms of methods, frequencies, and time of the assessment of SMM, which led to inconsistent results relating to the extent of skeletal muscle changes and its associated impact. Recently, a few prospective studies have revealed that SML is more common in the first few months following surgical GI cancer treatment, with a prevalence from 34^15^ to 82%.^12^ However, the patterns of change in SMM during this period are less understood.

Factors such as age, gender, and health behaviors (e.g., smoking and alcohol consumption, physical activities, and nutrition) are known to be linked with SMM.^16^ Among patients with cancer, factors such as advanced cancer stage and its treatment, comorbidities, reduced energy intake, and inflammatory response have also been shown to be associated with SML.^17–20^ However, few studies have investigated SML while simultaneously taking these factors into account. Hence, to address current gaps, the aims of this study were to (1) identify the patterns of change in SMM in the first 3 months following curative GI cancer surgery, (2) examine the relationship of patterns of change with patients’ QoL, and (3) explore predictors of postoperative SML.

## PATIENTS AND METHODS

We conducted a single-center study using a prospective cohort design.

### Setting and Participants

Using a consecutive sampling method, participants were recruited from the First Affiliated Hospital of Nanjing Medical University between September 2021 and May 2022. Study participants were adults (> 18 years of age) who were newly diagnosed with GI cancer and were to receive gastric, esophageal, or colorectal surgery as their primary treatment. Patients were excluded if they (1) had physical deformities or severe cardiopulmonary complications or current psychiatric illnesses, (2) were receiving palliative surgery, (3) were diagnosed with cancer cachexia at admission, or (4) were unavailable for follow-up. The trained nurses reviewed patients’ records and assessed their eligibility at admission. Eligible patients were invited to the study and those who expressed interest were referred to the first author (XX) for further study information and written consent. The consent process was completed before the surgery.

The sample size was first estimated on the basis of the prevalence of postoperative SML as reported in previous studies.^12,15^ The minimum sample size requirement was 345 assuming a prevalence of 34%. We also assumed there were at least two latent subgroups within the sample on the basis of the patterns of change in postoperative SMM. Growth mixture modeling was used to identify the subgroups, and a minimum of 200 participants was required for model construction.^21^ Assuming 10% of loss to follow up,^22^ the estimated sample size for this study was 384. Although recruitment reached the estimated sample size by February 2022, a decision was made to extend the recruitment period by 3 months to recruit as many participants as possible due to high dropout rate caused by coronavirus disease 2019 (COVID-19)-related restrictions at the participating site and nearby areas.

This study was approved by the Human Research Ethics Committees of the Nanjing Medical University (approval no. 2021-606) and the Queensland University of Technology (approval no. 2021-4223).

### Measurements and Data Collection

#### Skeletal Muscle Mass

Participants’ SMM was the primary outcome of this study, assessed by bioelectrical impedance analysis (BIA) using Inbody 270 (Biospace, Seoul, Korea) at four timepoints: T0 (at admission/the day before surgery), T1 (7 days after surgery/at discharge), T2 (1 month after surgery), and T3 (3 months after surgery). All measurements were carried out by the first author (XX) following the instructions of the manufacturer.

Skeletal muscle index (SMI) was calculated by dividing the appendicular SMM (kg) by the square of the body height (m^2^).^23^ Since the definition of normal SMI is gender specific (i.e., SMI ≥ 7.0 kg/m^2^ for male participants and ≥ 5.7 kg/m^2^ for female participants) according to the Asian Working Group for Sarcopenia (AWGS) 2019 criteria,^23^ we proposed a new measure, namely, derived skeletal muscle index (dSMI), to count the difference between individual SMI and the gender-specific norm. dSMI ≥ 0 indicates that the individual’s SMM is normal, while dSMI < 0 indicates that the individual has low SMM. In addition, low SMM, low muscle strength, and/or low physical performance were considered as sarcopenia.^23^ Low muscle strength was defined as handgrip strength < 28 kg for male participants and < 18 kg for female participants, while low physical performance was defined as a gait speed < 1 m/s.^23^ The methods of handgrip strength and gait speed measurements were followed as suggested by AWGS.^23^ The postoperative skeletal muscle change (ΔSMI) at each timepoint was calculated using the following equation:

$$\Delta {\text{SMI}}(\% ) = \frac{{{\text{SMI}}\,{\text{at}}\,{\text{Tx}}\,({\text{T1}}\,{\text{or}}\,{\text{T}}2\,{\text{or}}\,{\text{T}}3) - {\text{SMI}}\,{\text{at}}\,{\text{T}}0}}{{{\text{SMI}}\,{\text{at}}\,{\text{T}}0}} \times 100\% .$$

#### Postoperative Complications

Postoperative complications were regularly monitored and assessed by the participant’s treating specialists. We obtained the data from participants’ medical records. According to the Clavien–Dindo classification (CDC), the severity of postoperative complications was graded from I to V, with total complications classified as CDC > II and severe complications as CDC ≥ IIIa.

#### Patient-Reported Quality of Life

The simplified Chinese version of the European Organisation for Research and Treatment of Cancer (EORTC) Quality of Life Core Questionnaire (QLQ-C30) was used to evaluate health-related QoL, including one global scale, five functional scales, and eight symptom scales.^24^ All scales score from 0 to 100. A higher score for the global health status or functional scale represents better QoL or functioning; a higher score for a symptom scale indicates more severe symptoms. All participants were asked to complete the questionnaire at each time of the BIA test. If the participant was not able to attend the follow-up BIA tests at the hospital, the questionnaires were completed by telephone interview. Different modes of administration (i.e., telephone interview versus self-administration at the hospital) did not impact the reliability of the questionnaire.^25^

#### Predictors of Postoperative Skeletal Muscle Loss

A range of potential predictors of postoperative SML (as presented in Tables 1 and 2) was identified through literature reviews, including participants’ general demographic and clinical characteristics and relevant biomarkers. All data were obtained from their medical records at admission. Information in relation to GI surgery method, operation time, and intraoperative blood loss was also obtained from their medical records after surgery.

**Table 1:** Demographic and clinical characteristics of study participants at admission

Variables	Total (*n =* 483)	Completed muscle assessment at all time (*n =* 242, 50.1%)	Did not complete muscle assessment at all time (*n =* 241, 49.9%)	Differences between two groups
*Demographic characteristics*				
Age in years, mean ± SD	64.08 ± 9.25	64.22 ± 9.16	63.94 ± 9.36	*t* = −0.339, *p =* 0.735
Age groups				
< 50 years	35 (7.2%)	14 (5.8%)	21 (8.7%)	*χ*^2^ = 3.230, *p =* 0.664
50–54 years	41 (8.5%)	20 (8.3%)	21 (8.7%)	
55–59 years	65 (13.5%)	29 (12.0%)	26 (10.8%)	
60–64 years	110 (22.8%)	53 (21.9%)	57 (23.7%)	
65–69 years	113 (23.4%)	62 (25.6%)	51 (21.2%)	
> 69 years	119 (24.6%)	64 (26.4%)	55 (22.8%)	
Gender				
Male	390 (80.7%)	187 (77.3%)	203 (84.2%)	*χ*^2^ = 3.762, *p =* 0.052
Female	93 (19.3%)	55 (22.7%)	38 (15.8%)	
Education level				
Not complete high school	411 (85.1%)	212 (87.6%)	199 (82.6%)	*χ*^2^ = 2.409, *p =* 0.121
High school or above	72 (14.9%)	30 (12.4%)	42 (17.5%)	
Working status				
Retired/sick leave	402 (83.2%)	205 (84.7%)	197 (81.7%)	*χ*^2^ = 0.762, *p =* 0.383
Works full time/part time	81 (16.8%)	37 (15.3%)	44 (18.3%)	
Personal monthly income (CNY, ¥)				
< 3000	325 (67.3%)	171 (70.7%)	154 (63.9%)	*χ*^2^ = 2.507, *p =* 0.113
≥ 3000	158 (32.7%)	71 (29.3%)	87 (36.1%)	
Marital status				
Married/de facto	433 (89.6%)	214 (88.4%)	219 (90.9%)	*χ*^2^ = 0.776, *p =* 0.379
Single/divorced/widowed	50 (10.4%)	28 (11.6%)	22 (10.1%)	
Residence area				
Rural	275 (56.9%)	139 (57.4%)	146 (60.5%)	*χ*^2^ = 0.493, *p =* 0.483
Metropolitan	208 (43.1%)	103 (42.6%)	95 (39.4%)	
*Clinic characteristics*				
Cancer site				
Esophageal	60 (12.4%)	29 (12.0%)	31 (12.9%)	*χ*^2^ = 2.647, *p =* 0.266
Gastric	242 (50.1%)	130 (52.7%)	112 (46.5%)	
Colorectal	181 (37.5%)	83 (32.4%)	98 (40.7%)	
Cancer TNM staging				
I	91 (18.8%)	43 (17.8%)	48 (19.9%)	*U* = −1.785, *p =* 0.074
II	178 (36.9%)	81 (33.4%)	97 (40.3%)	
III	194 (40.2%)	106 (43.8%)	88 (36.5%)	
IV	20 (4.1%)	12 (5.0%)	8 (3.3%)	
Neoadjuvant therapy (NAT)				
No	452 (93.6%)	224 (92.6%)	228 (94.6%)	*χ*^2^ = 0.840, *p =* 0.359
Yes	31 (6.4%)	18 (7.4%)	13 (5.4%)	
Preoperative SMI, kg/m^2^	7.13 ± 0.90	7.08 ± 0.91	7.17 ± 0.88	*t* = 1.107, *p =* 0.269
Preoperative sarcopenia				
No	404 (83.6%)	199 (82.2%)	205 (85.1%)	*χ*^2^ = 0.707, *p =* 0.400
Yes	79 (16.4%)	43(17.8%)	36 (14.9%)	
Body mass index, BMI, kg/m^2^				
Normal weight (18.5 ~ < 24.0)	355 (75.3%)	172 (71.1%)	183 (75.9%)	*U* = −1.636, *p =* 0.102
Underweight (< 18.5)	43 (8.9%)	20 (8.3%)	23 (9.5%)	
Overweight or obese (≥ 24)	85 (17.6%)	50 (20.7%)	35 (14.5%)	
History of smoking				
Never	188 (38.9%)	91 (37.6%)	97 (40.2%)	*χ*^2^ = 0.359, *p =* 0*.*836
Previous smoker	229 (47.4%)	117 (48.3%)	112 (46.5%)	
Current smoker	66 (13.7%)	34 (14.0%)	32 (13.3%)	
History of drinking				
Never	206 (42.7%)	101 (41.7%)	105 (43.6%)	*χ*^2^ = 0.214, *p =* 0.899
Previous drinker	237 (49.1%)	120 (49.6%)	117 (48.5%)	
Current drinker	40 (8.3%)	21 (8.7%)	19 (7.9%)	
Comorbidity (e.g., diabetes, hypertension, stroke)				
No	314 (65.0%)	151 (62.4%)	163 (67.6%)	*χ*^2^ = 1.457, *p =* 0.228
Yes	169 (35.0%)	91 (37.6%)	78 (32.4%)	
Concurrent medication				
No	346 (71.6%)	163 (67.4%)	173 (71.8%)	*χ*^2^ = 1.119, *p =* 0.290
Yes	137 (28.4%)	79 (32.6%)	68 (28.2%)	
History of non-cancer related surgery				
No	306 (63.4%)	145 (59.9%)	161 (66.8%)	*χ*^2^ = 2.467, *p =* 0.116
Yes	177 (36.6%)	97 (40.1%)	80 (33.2%)	

The criteria of overweight/obesity are referred to the multidisciplinary clinical consensus on diagnosis and treatment of obesity (2021), which is proposed by the China Obesity Working Group and China diabetes Society

**Table 2:** Biomarkers of study participants at admission

Variables	Total (*n =* 483)	Completed muscle assessment at all time (*n =* 242, 50.1%)	Did not complete muscle assessment at all time (*n =* 241, 49.9%)	Differences between two groups
Serum calcium, mmol/L				
Normal (≥ 2.20)	237 (49.1%)	122 (50.4%)	115 (47.7%)	*χ*^2^ = 0.351, *p* = 0.554
Low (< 2.20)	246 (50.9%)	120 (49.6%)	126 (52.3%)	
Hemoglobin, g/L				
Normal (male ≥ 130, female ≥ 120)	301 (62.3%)	153 (63.2%)	148 (61.4%)	*χ*^2^ = 0.169, *p* = 0.681
Low (male<130, female<120)	182 (37.7%)	89 (36.8%)	93 (38.6%)	
Serum albumin, g/L				
Normal (≥ 35)	237 (49.1%)	123 (50.8%)	114 (47.3%)	*χ*^2^ = 0.600, *p* = 0.439
Low (< 35)	246 (50.9%)	119 (49.2%)	127 (52.7%)	
White blood cell count, WBC, 10^9^/L				
Normal (3.5–9.5)	426 (88.2%)	216 (89.3%)	210 (87.1%)	*U* = −0.129, *p* = 0.897
Low (< 3.5)	27 (5.6%)	12 (5.0%)	15 (6.2%)	
High (> 9.5)	30 (6.2%)	14 (5.8%)	16 (6.6%)	
Retinol binding protein, RBP, mg/L				
Normal (25.0–70.0)	400 (82.8%)	198 (81.8%)	202 (83.8%)	*χ*^2^ = 0.339, *p* = 0.560
Low (< 25.0)	83 (17.2%)	44 (18.2%)	39 (16.2%)	
Serum creatinine, μmol/L				
Normal (44–133)	472 (97.7%)	235 (97.1%)	237 (98.3%)	*χ*^2^ = 0.825, *p =* 0.364
Low (< 44) or high (> 133)	11 (2.3%)	7 (2.9%)	4 (1.7%)	
Prognostic nutritional index, PNI	47.35 (5.35)	47.7 (4.45)	47.3 (5.25)	*U* = −0.237, *p* = 0.812
Neutrophil-to-lymphocyte ratio, NLR	1.94 (1.50)	1.94 (1.39)	1.95 (1.50)	*U* = −0.126, *p* = 0.900
Platelet-to-lymphocyte ratio, PLR	118.27 (65)	119.23 (68.86)	115.82 (61.79)	*U* = −0.468, *p* = 0.640
Albumin-to-globulin ratio, AGR	1.46 (0.30)	1.47 (0.30)	1.46 (0.31)	*U* = −0.242, *p* = 0.809
Modified Glasgow prognostic score, mGPS				
0 (normal C-reactive protein and albumin level)	418 (86.5%)	207 (85.5%)	211 (87.6%)	U = −0.592, *p* = 0.554
1 (increased C-reactive protein only)	51 (10.6%)	29 (12.0%)	22 (9.1%)	
2 (increased C-reactive protein and hypoalbuminemia)	14 (2.9%)	6 (2.5%)	8 (3.3%)	
Controlling Nutritional Status Score, CONUT				
Normal (0–1)	248 (50.1%)	126 (52.1%)	116 (48.1%)	*U* = −0.674, *p* = 0.500
Mild malnutrition (2–4)	218 (45.1%)	103 (42.6%)	115 (47.7%)	
Moderate or severe malnutrition (5–12)	23 (4.8%)	13 (5.4%)	10 (4.1%)	
Carcinoembryonic antigen, CEA, ng/mL				
Normal (< 4.7)	353 (73.1%)	179 (74.0%)	174 (72.2%)	*χ*^2^ = 0.192, *p* = 0.661
High (≥ 4.7)	130 (26.9%)	63 (26.0%)	67 (27.8%)	
Cancer antigen 199, CA199, U/mL				
Normal (< 39)	435 (90.1%)	216 (89.3%)	219 (90.9%)	*χ*^2^ = 0.352, *p* = 0.553
High (≥ 39)	48 (9.9%)	26 (10.7%)	22 (9.1%)	
Nutrition Risk Screening 2002 (NRS2002) score				
< 3 (no risk of malnutrition)	401 (83.0%)	204 (84.3%)	197 (81.7%)	*χ*^2^ = 0.559, *p =* 0.455
≥ 3 (at risk of malnutrition)	82 (17.0%)	38 (15.7%)	44 (18.3%)	

The scoring methods of CONUT and NRS2002 referred to: Ignacio de Ulíbarri, J., et al., CONUT: a tool for controlling nutritional status. First validation in a hospital population. Nutricion Hospitalaria, 2005. **20**(1): p. 38-45, and Kondrup, J., et al., Nutritional risk screening (NRS 2002): a new method based on an analysis of controlled clinical trials. Clinical Nutrition (Edinburgh, Scotland), 2003. **22**(3): p. 321-336, respectively

### Statistical Analyses

Descriptive statistics were used to summarize baseline characteristics and muscle assessment at each timepoint by presenting means and standard deviations for normally distributed variables, median, and range for non-normally distributed continuous variables, and frequencies and percentages for categorical variables.

#### Patterns of Change in Skeletal Muscle Mass

Independent sample *t*-tests (for normally distributed measures) or Mann–Whitney *U* tests (for non-normally distributed measures) were performed to test the difference between postoperative SMI/dSMI and baseline SMI/dSMI, and SMI by cancer site at each timepoint. Pearson’s chi-squared tests were conducted to compare the prevalences of low skeletal muscle index (dSMI < 0), sarcopenia, and postoperative SML by cancer site at each timepoint. Growth mixture modeling (GMM) was used to examine heterogeneity among participants in relation to the patterns of change in postoperative SMM. dSMI was used as an indicator for SMM in GMM. The models were estimated using maximum likelihood estimator. The model fit was assessed using a combination of indicators: the smaller the value of Akaike information criterion (AIC) and Bayesian information criterion (BIC), the better the model fit. Entropy value above 0.8 was acceptable. When the Lo–Mendell–Rubin likelihood ratio test (LMR) and bootstrapped likelihood ratio test (BLRT) showed *p* < 0.05, *k* number of latent classes was viewed to be significantly better than *k*-1 classes.^26^ GMM was conducted using Mplus (version 8.0, Muthén & Muthén, USA).^27^ The number of the subgroups was identified on the basis of the characteristics of muscle change in each class. The demographic and clinical features of subgroups were then described and compared. Factors that showed a significant difference between subgroups were considered as potential predictors of postoperative SML.

#### Relationship Between Postoperative Skeletal Muscle Loss and Health Outcomes

The prevalences of total and severe postoperative complications in each identified subgroup were compared using a Pearson’s chi-squared test. Scores for each QLQ-C30 scale of the subgroups were compared at each timepoint. If two subgroups were identified, independent sample *t*-tests (for normally distributed measures) or Mann–Whitney *U* tests (for non-normally distributed measures) were performed. If multiple subgroups were identified, parametric (e.g., one-way analysis of variance) or nonparametric (e.g., Kruskal–Wallis *H* test) tests were applied. A post hoc test was conducted to identify which groups differed from each other.

#### Predictors of Postoperative Muscle Loss

The correlations between potential predictors were explored to check collinearity. Highly correlated potential predictors were removed on the basis of theoretical reasons before being included in the next logistic regression analysis. Univariate logistic regression analysis was conducted first to explore the relationship between each potential predictor and SML. Then, a forward stepwise method was used to construct a multiple logistic regression model and identify independent predictors of SML. The predictive ability was assessed using the area under ROC curve (AUC). The higher the AUC value, the better its ability to distinguish between muscle loss and non-muscle loss groups.

All participants had observations at baseline. However, some participants had missing data on the primary outcome (i.e., SMM) at follow-up. Rates of missing data at each timepoint were calculated, and the reasons and patterns of missing data were investigated. We compared the characteristics of participants who completed muscle assessment at all timepoints with those who did not. Assuming data were missing at random, multiple imputation was applied using chained equations. For the participants who had missing SMM at follow-up, the SMM of the one previous timepoint and other covariates (i.e., age, gender, stage of cancer, and cancer site) were used to impute the missing values. The number of imputed datasets was based on the percentage of missing data at T3, as suggested by Van Buuren.^28^ For example, if the rate of missing data was 30% at T3, 30 imputed datasets would be generated. The matrix and seed were set using the default settings. Results of imputed datasets were pooled using Rubin’s rules.^29^ The imputation was conducted in R studio (version 4.1.3) using the Multivariate Imputation by Chained Equations (MICE) package. Further analysis was performed to compare whether there were differences between completed cases and imputed values in relation to baseline characteristics and QoL of the participants, and patterns of change in SMM. If the results of complete cases and imputed values were qualitatively the same, only the findings from complete cases analysis were reported.

All analyses were conducted in SPSS (version 26.0) unless specified. A two-tailed *p* value < 0.05 was considered statistically significant for all tests in this study.

## RESULTS

### Recruitment of Participants and Loss to Follow-Up

**Fig. 1:**
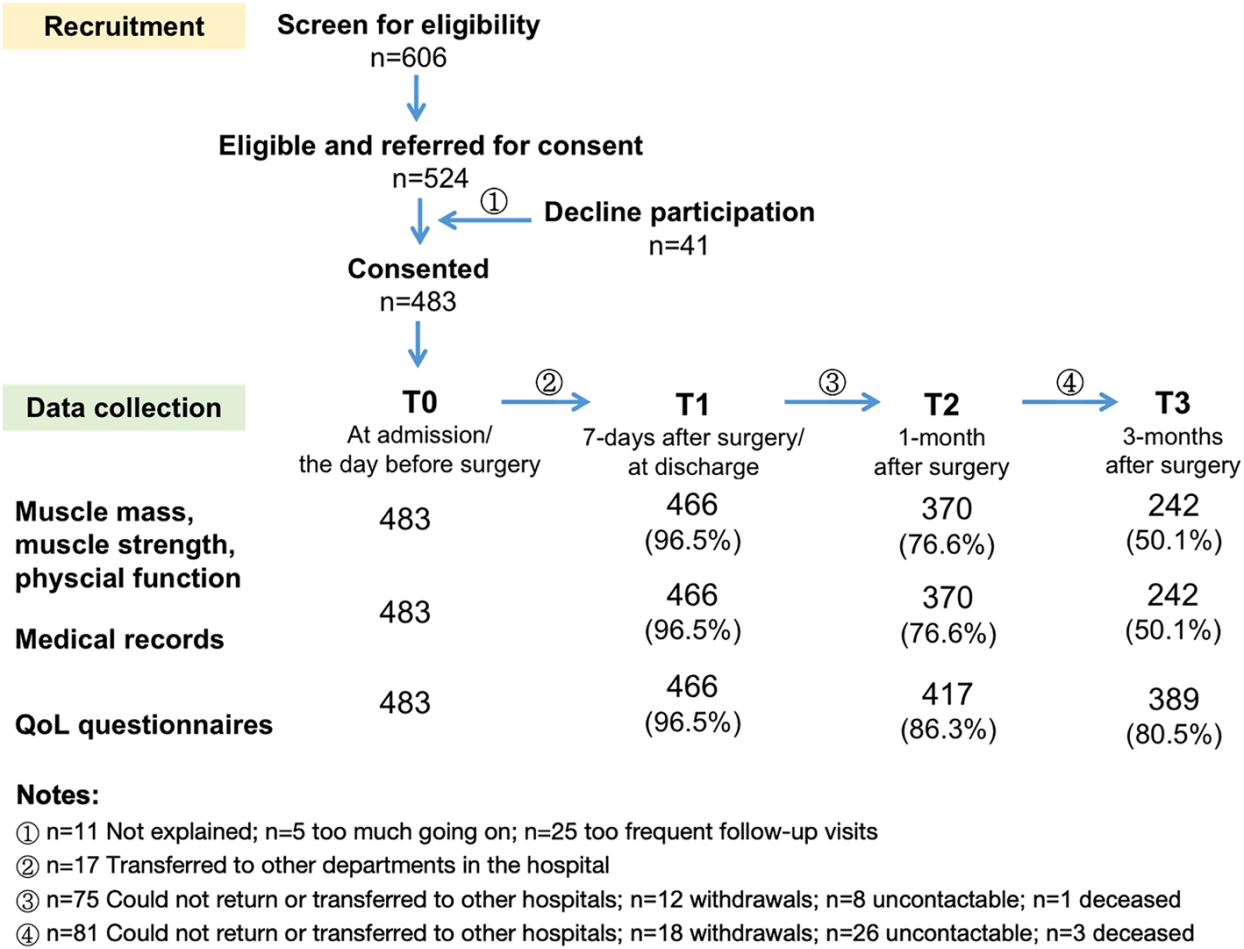
Recruitment of study participants and data collection flowchart

During the recruitment period, a total of 606 patients were admitted to the participating departments and subsequently referred for eligibility screening. Of the 524 eligible patients, 483 patients (92.2%) consented to the study. All participants completed the muscle assessments and QoL questionnaire before surgery, 242 (50.1%) completed the muscle assessment, and 389 (80.5%) completed the questionnaire at all timepoints after surgery. Figure 1 summarizes the number of participants at each timepoint and reasons for loss to follow-up. The rate of missing data regarding muscle assessment was 3.5%, 23.4%, and 49.9% at T1, T2, and T3, respectively. The pattern of missing data was monotonic. Considering the main reasons for loss to follow-up, the missing data regarding SMM was likely missing at random.

### Characteristics of Study Participants

Table 1 presents the demographic and clinical characteristics of the study participants at admission. There were no significant differences between participants who had complete data and those who did not in terms of baseline demographic and clinical characteristics. Table 2 presents the biomarkers collected at admission. There were no significant differences between the two groups. Most participants (73.1%, *n* = 353) received minimally invasive surgery (e.g., laparoscopic, robotic surgery). The proportion of the participants who received minimally invasive surgery was significantly (*p =* 0.043) lower in those who had complete data (69.0%, 167 out of 242) than those who did not (77.2%, 186 out of 241). No significant differences were observed between the two groups regarding surgery time (*p* = 0.088) and intraoperative blood loss (*p* = 0.117).

### Patterns of Change in Skeletal Muscle Mass in the First 3 Months after Surgery

The mean SMI was 6.96 ± 0.84, 6.67 ± 0.93, 6.62 ± 1.02 kg/m^2^, and the median ΔSMI was − 2.5% (− 11.0~9.8%), − 5.3% (− 26.0 to 4.0%), and − 5.6% (− 30.1 to 5.5%), at T1, T2, and T3, respectively. The median ΔSMI was negative at each timepoint, indicating postoperative skeletal muscle loss. Only 19 out of 242 participants (7.9%) did not experience SML at any timepoint in the first 3 months following surgery. The median dSMI was 0.3 (− 1.7 to 2.5), 0.2 (− 1.5 to 2.5), − 0.1 (− 1.7 to 2.3), and − 0.2 (− 1.9 to 2.2) at T0, T1, T2, and T3, respectively. The prevalence of low SMM (dSMI < 0) was 21.3%, 33.5%, 54.1%, and 53.3%, and the prevalence of sarcopenia was 17.2%, 21.0%, 33.0%, and 31.8%, at T0, T1, T2, and T3, respectively.

Significant differences were observed between cancer sites regarding the mean SMI, prevalence of low SMM, and prevalence of sarcopenia at T0, T1, and T2, respectively (see Supplementary S1). However, there was no significant difference in the prevalence of postoperative SML between cancer sites.

Table 3 presents model fitting results for different numbers of latent classes. On the basis of the values of AIC and BIC, a four-class model was a better solution. The entropy for the four-class model was acceptable. Compared with the four-class model, the five-class model did not significantly improve the model fit (LMR: *p* = 0.489). As such, a four-class model was identified as the best model.

**Table 3:** Model fitting statistics and estimation

Number of latent classes	AIC	BIC	Entropy	LMR	BLRT	Proportion of each class
2	712.004	750.382	0.755	0.028	< 0.001	0.738/0.261
3	683.338	735.672	0.728	0.037	< 0.001	0.224/0.448/0.329
4	650.755	713.556	0.830	0.035	< 0.001	0.161/0.231/0.272/0.335
5	679.758	749.537	0.798	0.489	0.010	0.321/0.194/0.187/0.004/0.294

*AIC* Akaike information criterion, *BIC* Bayesian information criterion, *LMR* Lo–Mendell–Rubin likelihood ratio test, *BLRT* bootstrapped likelihood ratio test

**Fig. 2:**
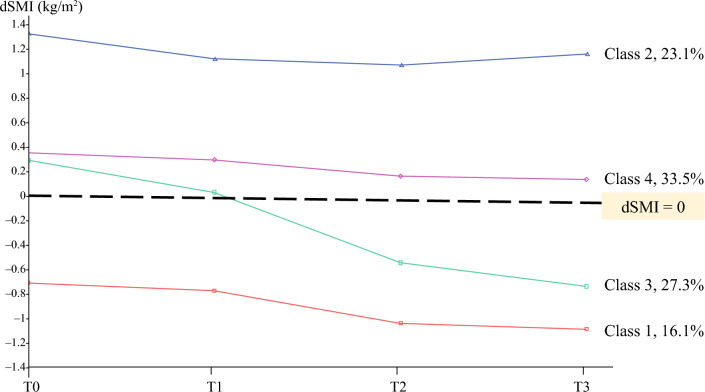
Latent class model for derived skeletal muscle index (dSMI) in the first 3 months after surgery (*n* = 242); percentages indicate the percentages of participants each class accounts for; T0 at admission/the day before surgery, T1 7 days after surgery/at discharge, T2 1 month after surgery, T3 3 months after surgery, dSMI derived skeletal muscle index, dSMI = 0 indicates the SMI was 5.7 kg/m^2^ for female patients or 7 kg/m^2^ for male patients

Figure 2 illustrates the four latent classes that emerged from the GMM. Participants in class 1 (*n* = 39, 16.1%) had low SMM before surgery and experienced further SML after surgery. Although participants in class 2 (*n* = 56, 23.1%) experienced SML at 7 days and 1 month after surgery, their SMM was above normal at all times and showed a recovery trend at 3 months after surgery. Participants in class 3 (*n* = 66, 27.3%) had a SMM just above normal before surgery, but experienced consistent SML after surgery. A sharp decline was observed at 1 month after surgery in particular. Class 4 was the largest group, including 33% of the total participants (*n* = 88). Their SMM was just above normal and remained at a similar level throughout the study period. We also identified the best model using the imputed dataset. The pattern and proportion of each class were similar to the results using the complete cases (Supplementary S2).

On the basis of the above results, three participant subgroups were identified. Classes 2 and 4 were considered as the mild SML group. Class 1 was regarded as moderate SML group and Class 3 was categorized as severe SML group. The profiles of the three subgroups are summarized in Supplementary S3. Compared with those in the moderate and severe SML subgroups, a higher percentage of the participants in the mild SML subgroup were below the age of 65, at work, living in metropolitan areas, diagnosed with early stage cancer, and without any comorbidities or current medications. A higher proportion of the participants in the mild SML subgroup received minimally invasive surgery and had a shorter operation time and less intraoperative blood loss than those in the moderate and severe SML subgroups. Moreover, there were significant differences in some blood-based biomarkers among subgroups. For instance, a higher proportion of the participants in the moderate and severe SML subgroups had low serum albumin, low PNI, and high platelet-to-lymphocyte ratio (PLR) than those in the mild SML subgroup.

### Relationship Between Postoperative Skeletal Muscle Loss and Health Outcomes

About 19.8% of the participants (*n* = 48) experienced at least one complication (CDC > II), and 7.0% (*n* = 17) had severe complications (CDC ≥ IIIa) within the first 3 months after surgery. The prevalences of total and severe complications were 10.9% and 2.9% in the mild SML subgroup, 25.6% and 12.8% in the moderate SML subgroup, and 34.8% and 12.1% in the severe SML subgroup. Participants with moderate/severe SML experienced significantly higher prevalences of total (*p* < 0.001) and severe (*p* = 0.016) complications than those with mild SML.

**Fig. 3:**
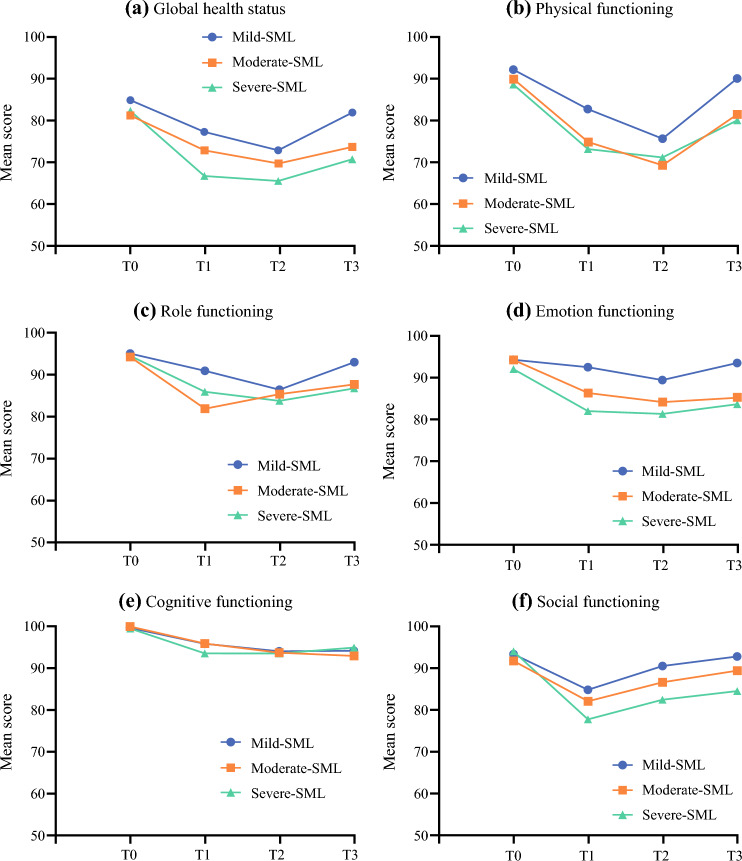
Changes in QLQ-C30 global health status and functioning scales over time (*n* = 242); scores range from 0 to 100, a higher score represents better QoL or functioning; T0 at admission/the day before surgery, T1 7 days after surgery/at discharge, T2 1 month after surgery, T3 3 months after surgery

The mean scores of QLQ-C30 by participant subgroups at each timepoint are summarized in Supplementary S4. Figure 3 shows the changes in global health status and functioning scales over time by participant subgroups. Overall, participants in the moderate and severe SML subgroups reported significantly lower QoL regarding global health status and physical function than those in the mild SML subgroup at all timepoints. Additionally, participants in these two subgroups reported worse role, emotional, and social functioning, and more severe symptoms, such as fatigue, pain, and insomnia, than those in the mild SML subgroup after surgery.

### Predictors of Postoperative Muscle Loss

We combined the moderate SML subgroup and severe SML subgroup and explored predictors of moderate/severe postoperative SML. Working status, concurrent medication, operation time, intraoperative blood loss, serum albumin, and BMI were excluded after multicollinearity testing (Supplementary S5). A total of 14 variables was entered into the univariate logistic regression models (Supplementary S6) and binary logistic regression models. Table 4 presents the results of binary logistic regression for predictors of moderate/severe postoperative muscle loss. The results demonstrated that advanced age, preoperative sarcopenia, low prognostic nutrition index, and advanced cancer stage were independent predictors for moderate/severe postoperative muscle loss. The model had a good predictive ability with an overall prediction accuracy of 83.1% and a prediction accuracy of 81.9% for moderate/severe skeletal muscle loss group. The AUC was 0.896, 95% CI (0.854, 0.937).

**Table 4:** Binary logistic regression for predictors of moderate/severe postoperative* muscle loss*

Variables	B	SE	*P*	OR	95% CI
Age ≥ 65 years	1.320	0.357	< 0.001	3.743	1.858–7.542
Preoperative sarcopenia	2.476	0.563	< 0.001	11.896	3.942–35.895
Prognostic nutrition index (PNI) ≤ 45	2.034	0.394	< 0.001	7.646	3.534–16.539
TNM staging (III, IV)	1.623	0.357	< 0.001	5.070	2.517–10.209
Constant	− 7.807	1.030	< 0.001	–	–

Hosmer–Lemeshow goodness-of-fit test: *χ*^2^ =13.664, *p* = 0.091. The values of − 2 Log Likelihood, Cox and Snell *R*-squared, and Nagelkerke *R*-squared were 201.999, 0.414, and 0.555, respectively.

*B* coefficient for the constant, *SE* standard error, *OR* odds ratio, *CI* confidence interval

## DISCUSSION

In this study, we examined changes in SMM over the first 3-month period following GI cancer surgery. To our knowledge, this study is the first to identify the patterns of muscle change and heterogeneity in patterns during this period. We found the majority (92%) experienced SML after GI cancer surgery, with three distinct patterns of postoperative muscle change identified among the study participants. Specifically, more than half (57%) of the participants experienced mild postoperative SML and had normal SMM throughout the study. About 16% of the participants had low SMM before surgery and experienced further moderate SML after surgery. Notably, another 27% of the participants had normal SMM before surgery but experienced consistent and severe SML after surgery. Given the higher prevalence of preoperative sarcopenia among patients with GI cancer than other cancer populations,^30^ current research and clinical practice is often focused on patients with preoperative sarcopenia. Most participants in our study (84%) had a normal SMM before surgery, and as such the subgroup comprising 27% of the sample who had normal SMM before surgery but experienced severe loss after surgery is most likely overlooked. As postoperative SMM is not routinely assessed, this subgroup is likely to remain unnoticed. Our results highlight the importance of recognizing different patterns of postoperative muscle change and assessment and monitoring of SMM after GI cancer surgical treatment.

Consistent with previous studies,^17,18,31^ our results showed that participants with upper GI cancer, especially esophageal cancer, had significantly lower SMM and a higher prevalence of sarcopenia compared with those with colorectal cancer at all timepoints. However, there was no significant difference in terms of the prevalence of postoperative SML stratified by cancer site, indicating that patients with both upper GI and colorectal cancer were vulnerable to SML after surgery. The median SML at 1 month and 3 months in this study was similar to previous studies.^32,33^ However, the median SML at 7 days in previous studies has been reported to exceed 4%,^12,17^ which was higher than our findings. This could be related to the different clinical practices among countries. Approximately 80% of the participants in other studies received neoadjuvant therapy (NAT), with NAT found to be a risk factor for both preoperative and postoperative SML.^34^ Currently, NAT has not yet been widely adopted in China,^35^ and only 6% of the participants received it in our study. Our findings further highlight the importance of assessing and monitoring SMM both before and for a period of time after the surgical treatment.

There is some evidence that low SMM could be associated with poor health-related QoL and physical function in patients with cancer.^36^ However, very few studies have investigated the impact of SML on QoL in patients with cancer. One study found that patients with SML ≥ 10% at 7 days after GI cancer surgery experienced worse QoL at 1 month and 3 months after surgery.^18^ Instead of assessing SML and QoL separately at different timepoints, we monitored the changes in SMM and QoL concurrently in our study. Our results revealed that patients who had moderate/severe SML experienced persistent poorer general health, worse physical functioning, and more symptom burden than those who had a mild SML. It is essential to manage and mitigate SML in postoperative care as this will likely contribute to overall improvements in patients’ well-being.

Given the negative impact of SML on patients’ QoL, early identification of those who are at risk of poor health outcomes is critical. We found that advanced age (65 years or older), preoperative sarcopenia, inflammation (as indicated by a low PNI), and advanced cancer stage (III and IV) were independent predictors of moderate/severe SML in the first 3 months after GI cancer surgery. These findings are consistent with previous studies that have investigated SML after gastrectomy for gastric cancer, where advanced age was identified as a risk factor.^18,37^ Additionally, the association between preoperative sarcopenia and advanced cancer stage with SML has been consistently reported in studies of people with colorectal cancer.^3^ Systemic inflammatory responses have been reported to be associated with skeletal muscle wasting among GI cancer patients.^38^ Although we tested a range of blood-based biomarkers in this study, including systemic inflammation indicators such as PNI, neutrophil-to-lymphocyte ratio (NLR), PLR, and modified Glasgow prognostic score (mGPS), only a low PNI was found to be an independent predictor of moderate/severe SML. Future studies are required to further investigate the underlying mechanism of postoperative SML and validate the predictive value of PNI. Contrary to some studies,^18–20^ comorbidities and surgical method were not independent predictors of moderate/severe postoperative SML in this study.

A key strength of this study was the adoption of a prospective design. Assessments of SMM in previous retrospective studies were typically reported after the first 3 months. Such an approach could miss the opportunity for timely management of SML and its associated symptom burden. Moreover, previous studies have assumed that changes in SMM among patients in the first 3 months are homogeneous, potentially overlooking patients who are at risk of moderate or severe SML and poor health outcomes. The prospective collection of a range of demographic and clinical characteristics of the participants and relevant biomarkers enabled this study to provide a more thorough exploration of potential predictors of SML. These data often are not available in retrospective studies yet have important implications in the management of postoperative SML.

This study has several limitations. First, the generalizability of our results was limited since the study was conducted at a single health center in China. The characteristics of patients and clinical practices at the participating site could differ from other sites. Nonetheless, our participants are similar to the general Chinese cancer population as shown in the Chinese National Cancer Registry in terms of gender, age, and social economic status.^39^ The study faced challenges with substantial missing data for the muscle outcome measure due to the impact of COVID-19. While this could impact both the generalizability and validity of our results, we confirmed that the complete cases were similar to the incomplete cases in terms of baseline demographic and clinical characteristics. However, the proportion of the participants who received minimally invasive surgery was significantly lower in those who had complete data. Although the operation method was not shown to be an independent predictor of moderate/severe SML, this difference might have led to an overestimation of postoperative SML. To further explore the impact of the high loss to follow-up rate, we applied multiple imputation including covariates such as age and stage of cancer in the imputation model. The results obtained using the imputed data were the same as that using the complete cases. As such, our overall findings from the completed cases likely represent the overall sample recruited to the study. The second limitation of this study is that we used BIA to measure SMM Although the CT method is considered to be the gold standard, BIA is also recommended by the AWGS due to its cost-effectiveness, noninvasiveness, and availability.^25^ Another limitation is that we did not assess participants’ nutritional status (i.e., energy intake) and physical activities due to the complexity and required resources. These factors are known to be closely linked to SMM and functioning. Future studies that include direct assessments of these factors alongside other risk factors would further our understanding of the underlying mechanism of postoperative SML.

This study has significant implications for both clinical practice and research. Health professionals need to enhance their awareness and knowledge concerning postoperative SML associated with surgical GI cancer treatment. They should consider incorporating regular assessments of SMM and function as part of routine practice and as early as at 7 days and 1 month after surgery. Patient-reported quality of life measurement should also be incorporated into routine practice given the relationship between quality of life and SML. Future research is required to explore how subgroups identified in this study differ in terms of survival and other health outcomes in the long term. To better understand the underlying mechanism of SML, it is also important to explore nutrition- and physical-activity-related factors that were not included in this study. The findings of this study also provide an important base to inform the development of suitable intervention programs to actively manage SML and enhance the overall well-being of patients.

## CONCLUSIONS

We have demonstrated that SML is common in the first 3 months following GI cancer surgery. However, patterns of muscle change vary greatly among patients. Patients with an advanced age (≥ 65), preoperative sarcopenia, advanced cancer, and inflammation are especially at risk of moderate/severe SML and poor quality of life. Early identification of these patients and ongoing monitoring and management of the condition is critical to improve their health outcomes.

## Supplementary Information

Below is the link to the electronic supplementary material.

Supplementary file1 (DOCX 445 KB)
