# Supplementary material for: Changes in Skeletal Muscle Mass in the First 3 Months Following Gastrointestinal Cancer Surgery: A Prospective Study
Source: Ann Surg Oncol. 2024 Sep 4;31(13):8651–63. doi: 10.1245/s10434-024-16109-8 (PMC11549114; doi:10.1245/s10434-024-16109-8)
Supplement: Supplementary file 1 — Supplementary file1 (DOCX 445 KB) [file 10434_2024_16109_MOESM1_ESM.docx]

**Supplementary S1 Skeletal muscle index and prevalences of low skeletal muscle index, sarcopenia, and postoperative skeletal muscle loss stratified by cancer site**

**Table A** Skeletal muscle index (SMI) stratified by cancer site

| **Timepoint** | **Esophageal cancer** | | **Gastric cancer** | | **Colorectal cancer** | | **Difference by cancer site at each time point** |
| --- | --- | --- | --- | --- | --- | --- | --- |
|  | Valid *n* | SMI | Valid *n* | SMI | Valid *n* | SMI |  |
| T0 | 60 | 6.86 ± 0.88 | 242 | 6.92 ± 0.90 | 181 | 7.51 ± 0.78 | **F = 27.520, *p < .*001** |
| T1 | 52 | 6.70 ± 0.80 | 239 | 6.79 ± 0.85 | 175 | 7.29 ± 0.75 | **F = 26.510, *p < .*001** |
| T2 | 41 | 6.43 ± 0.80 | 187 | 6.49 ± 0.91 | 142 | 7.06 ± 0.87 | **F = 27.821, *p < .*001** |
| T3 | 29 | 6.41 ± 0.85 | 130 | 6.36 ± 0.99 | 83 | 7.09 ± 0.98 | **F = 15.227, *p < .*001** |

**Table B** Prevalence of low skeletal muscle index (dSMI < 0) stratified by cancer site

| **Timepoint** | **Esophageal cancer** | | **Gastric cancer** | | **Colorectal cancer** | | **Difference by cancer site at each time point** |
| --- | --- | --- | --- | --- | --- | --- | --- |
|  | Valid *n* | *n* (%) | Valid *n* | *n* (%) | Valid *n* | *n* (%) |  |
| T0 | 60 | 20 (33.3) | 242 | 59 (24.3) | 181 | 24 (13.3) | **χ2 = 13.521, *p = .*001** |
| T1 | 52 | 27 (51.9) | 239 | 86 (36.0) | 175 | 43 (24.6) | **χ2 = 14.852, *p < .*001** |
| T2 | 41 | 29 (70.7) | 187 | 105 (56.1) | 142 | 66 (46.5) | **χ2 = 8.203, *p = .*017** |
| T3 | 29 | 20 (69.0) | 130 | 72 (55.4) | 83 | 37 (44.6) | χ2 = 5.623, *p = .*060 |

**Table C** Prevalence of sarcopenia stratified by cancer site

| **Timepoint** | **Esophageal cancer** | | **Gastric cancer** | | **Colorectal cancer** | | **Difference by cancer site at each time point** |
| --- | --- | --- | --- | --- | --- | --- | --- |
|  | Valid *n* | *n* (%) | Valid *n* | *n* (%) | Valid *n* | *n* (%) |  |
| T0 | 60 | 16 (26.7) | 242 | 51 (21.1) | 181 | 16 (8.8) | **χ2 = 15.220, *p < .*001** |
| T1 | 52 | 20 (38.5) | 239 | 59 (24.3) | 175 | 19 (10.9) | **χ2 = 22.343, *p < .*001** |
| T2 | 41 | 22 (53.7) | 187 | 69 (36.9) | 142 | 31 (21.8) | **χ2 = 17.218, *p < .*001** |
| T3 | 29 | 13 (44.8) | 130 | 45 (34.6) | 83 | 19 (22.9) | χ2 = 5.780, *p = .*056 |

**Table D** Prevalence of postoperative skeletal muscle loss (SML) stratified by cancer site

| **SML** | **Timepoint** | **Esophageal cancer** | | **Gastric cancer** | | **Colorectal cancer** | | **Difference by cancer site at each time point** |
| --- | --- | --- | --- | --- | --- | --- | --- | --- |
|  |  | Valid *n* | *n* (%) | Valid *n* | *n* (%) | Valid *n* | *n* (%) |  |
| ΔSMI > 5% | T1 | 52 | 9 (17.3) | 239 | 42 (17.6) | 175 | 24 (13.7) | χ2 = 1.178, *p = .*555 |
|  | T2 | 41 | 21 (51.2) | 187 | 98 (52.4) | 142 | 78 (54.9) | χ2 = 0.282, *p = .*868 |
|  | T3 | 29 | 16 (55.2) | 130 | 73 (56.2) | 83 | 40 (48.2) | χ2 = 1.336, *p = .*513 |
| ΔSMI > 10% | T1 | 52 | 1 (1.9) | 239 | 2 (0.8) | 175 | 0 | χ2 = 2.604, *p = .*272 |
|  | T2 | 41 | 9 (22.0) | 187 | 47 (25.1) | 142 | 26 (18.3) | χ2 = 2.180, *p = .*336 |
|  | T3 | 29 | 7 (24.1) | 130 | 36 (27.7) | 83 | 28 (33.7) | χ2 = 1.322, *p = .*516 |

Notes: T0 = at admission/the day before surgery, T1 = 7-day after surgery/at discharge, T2 = 1-month after surgery, and T3 = 3-month after surgery.

**Supplementary S2 Growth Mixture Modelling using imputed muscle mass value at T1, T2, T3**

**Table A** presents model fitting results for different numbers of latent classes using imputed muscle mass value at T1, T2, and T3. A 4-class model was identified as the best model. **Figure A** presents the four latent classes that emerged from the GMM based on the imputed muscle mass value. Table B presents the comparison of the results using complete cases and imputed value. There was no significant difference in terms of the proportion of each class.

**Table A** Model fitting results for different numbers of latent classes using imputed muscle mass value at T1, T2, and T3

| **Number of latent classes** | **AIC** | **BIC** | **Entropy** | **LMR** | **BLRT** | **Proportion of each class** |
| --- | --- | --- | --- | --- | --- | --- |
| 2 | 1296.245 | 1346.406 | 0.724 | <0.001 | <0.001 | 0.435/0.565 |
| 3 | 1265.798 | 1328.498 | 0.792 | 0.009 | <0.001 | 0.185/0.462/0.353 |
| 4 | 1328.495 | 1403.735 | 0.788 | 0.0048 | <0.001 | 0.151/0.229/0.324/0.296 |
| 5 | 1274.591 | 1362.371 | 0.788 | 0.500 | 0.500 | 0/0.436/0.267/0.365/0.032 |

Notes: AIC = Akaike information criterion, BIC = Bayesian information criterion, LMR = Lo-Mendell-Rubin likelihood ratio test, BLRT = Bootstrapped likelihood ratio test


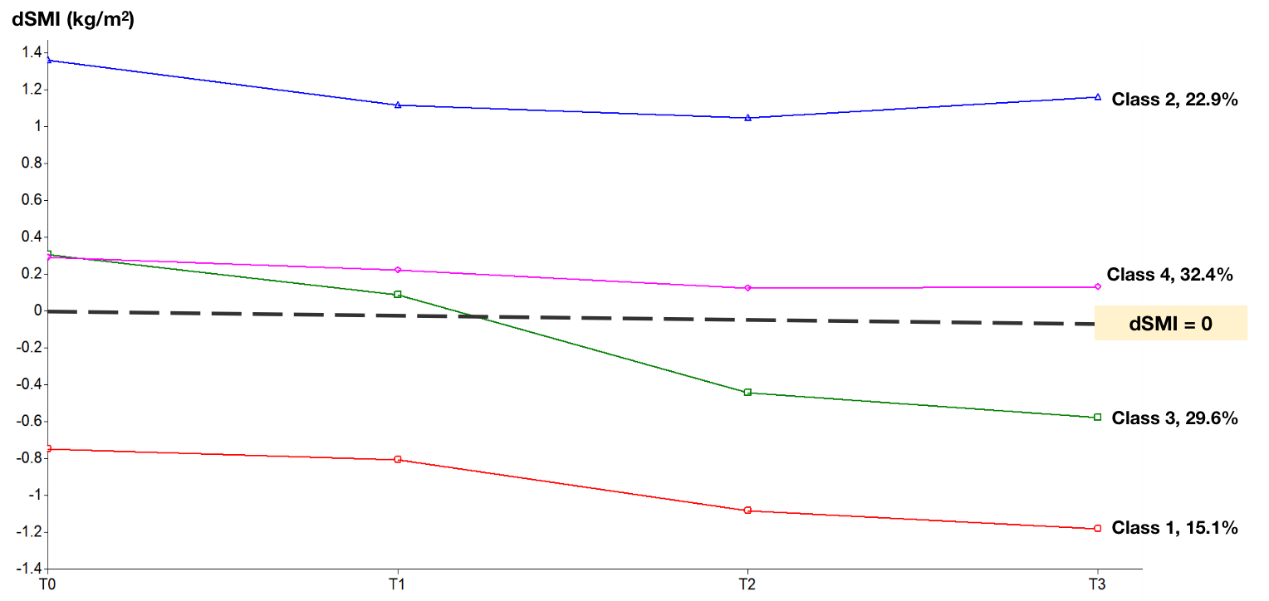
Notes: T0 = at admission/the day before surgery, T1 = 7-day after surgery/at discharge, T2 = 1-month after surgery, and T3 = 3-month after surgery. The percentages indicate the percentages of participants each class accounts for. dSMI = derived skeletal muscle index.

**Figure A** Latent class model for derived skeletal muscle index (dSMI) from baseline to three months after surgery (n*=*483)

**Table B** Comparison of the proportion of each latent class between the two GMM models

| **Latent class** | **Imputed value (n=483)** | **Complete cases (n=242)** | **Difference between each latent class** |
| --- | --- | --- | --- |
|  | *n* (%) | *n* (%) |  |
| 1 | 73 (15.1) | 39 (16.1) | χ2 = 0.124, *p =* 0.725 |
| 2 | 111 (22.9) | 56 (23.1) | χ2 = 0.408, *p =* 0.523 |
| 3 | 143 (29.6) | 66 (27.3) | χ2 = 0.428, *p =* 0.513 |
| 4 | 156 (32.4) | 81 (33.5) | χ2 = 0.101, *p =* 0.751 |

**Supplementary S3 Profiles of study participant subgroups**

| **Variables** | **Mild-SML**  **(*n =* 137, 56.6%)** | | **Moderate-SML**  **(*n =* 39, 16.1%)** | **Severe-SML**  **(*n =* 66, 27.3%)** | **Difference between subgroups** | |
| --- | --- | --- | --- | --- | --- | --- |
| **Demographic characteristics** | | | | | | |
| **Age, years** |  |  | |  |  |  |
| < 65 | 87 (63.5%) | 9 (23.1%) | | 20 (30.3%) | **χ2 = 31.183,**  ***p <* 0 *.*001** | |
| ≥ 65 | 50 (36.5%) | 30 (76.9%) | | 46 (69.7%) |  |  |
| **Gender** |  |  | |  |  |  |
| Male | 102 (74.5%) | 34 (87.2%) | | 51 (77.3%) | χ2 = 2.800, *p =* 0.247 | |
| Female | 35 (25.5%) | 5 (12.8%) | | 15 (22.7%) |  |  |
| **Education level** |  |  | |  |  |  |
| Not complete high school | 118 (86.1%) | 32 (82.1%) | | 62 (93.9%) | χ2 = 3.820, *p =* 0.148 | |
| high school or above | 19 (13.9%) | 7 (17.9%) | | 4 (6.1%) |  |  |
| **Working status** |  |  | |  |  |  |
| Retired/sickness | 105 (76.6%) | 35 (89.7%) | | 64 (97.0%) | **χ2 = 14.946,**  ***p <* 0.001** | |
| Work full-time/part-time | 32 (23.4%) | 4 (10.3%) | | 2 (3%) |  |  |
| **Personal monthly income (CNY, ¥)** |  |  | |  |  |  |
| < 3000 | 103 (75.2%) | 23 (59.0%) | | 45 (68.2%) | χ2 = 4.112, *p =* 0.128 | |
| ≥ 3000 | 34 (24.8%) | 16 (41.0%) | | 21 (31.8%) |  |  |
| **Marital status** |  |  | |  |  |  |
| Married/de facto | 127 (92.7%) | 32 (82.1%) | | 55 (83.3%) | χ2 = 5.669, *p =* 0.059 | |
| Single/divorced/widowed | 10 (7.3%) | 7 (17.9%) | | 11 (16.7%) |  |  |
| **Residence area** |  |  | |  |  | |
| Rural | 65 (47.4%) | 27 (69.2%) | | 47 (71.2%) | **χ2 = 12.937,**  ***p =*0*.*002** | |
| Metropolitan | 72 (52.6%) | 12 (30.8%) | | 19 (28.8%) |  |  |
| **Clinical characteristics** | | | | | | |
| **Cancer site** |  |  | |  |  |  |
| Esophageal cancer | 11 (8.0%) | 9 (23.1%) | | 9 (13.6%) | **χ2 = 9.972, *p =* 0*.*041** | |
| Gastric cancer | 74 (54.0%) | 23 (59.0%) | | 33 (50.0%) |  |  |
| Colorectal cancer | 52 (38.0%) | 7 (17.9%) | | 24 (36.4%) |  |  |
| **TNM staging** |  |  | |  |  |  |
| Ⅰ, II | 95 (69.3%) | 10 (25.6%) | | 18 (27.3%) | **χ2 = 43.342,**  ***p <* 0.001** | |
| Ⅲ, IV | 42 (30.7%) | 29 (74.4%) | | 48 (72.7%) |  |  |
| **Operation method** |  |  | |  |  |  |
| Open surgery | 21 (15.3%) | 27 (69.2%) | | 28 (42.4%) | **χ2 = 46.060,**  ***p <*0.001** | |
| Minimally invasive surgery | 116 (84.7%) | 12 (30.8%) | | 38 (57.6%) |  |  |
| **Neoadjuvant therapy, NAT** |  |  | |  |  |  |
| No | 127 (92.7%) | 34 (87.2%) | | 64 (97.0%) | χ2 = 3.633, *p =* 0.163 | |
| Yes | 10 (7.3%) | 5 (12.8%) | | 2 (3.0%) |  |  |
| **Operation time, min** |  |  | |  |  |  |
| ≤200 | 112 (81.8%) | 20 (51.3%) | | 45 (68.2%) | **χ2 = 15.483,**  ***p <* 0.001** | |
| > 200 | 25 (18.2%) | 19 (48.7%) | | 21 (31.8%) |  |  |
| **Intraoperative blood loss, mL** |  |  | |  |  |  |
| ≤100 | 130 (94.9%) | 31 (79.5%) | | 62 (93.9%) | **χ2 = 10.358,**  ***p =* 0.006** | |
| > 100 | 7 (5.1%) | 8 (20.5%) | | 4 (6.1%) |  |  |
| **Preoperative SMI** | 7.44±0.86 | 6.07±0.59 | | 6.95±0.66 | ***F*=48.558, *p <* 0 *.*001** | |
| **Preoperative sarcopenia** |  |  | |  |  |  |
| No | 131 (95.6%) | 8 (20.5%) | | 59 (89.4%) | **χ2 = 118.622,**  ***p <* 0 *.*001** | |
| Yes | 6 (4.4%) | 31 (79.5%) | | 7 (10.6%) |  |  |
| **BMI, kg/m^2^** |  |  | |  |  |  |
| Normal (18.5 ~ < 24.0) | 107 (78.1%) | 23 (59.0%) | | 42 (63.6%) | **H *=* 8.328^a^, *p =* 0.016** | |
| Under weight (< 18.5) | 0 | 13 (33.3%) | | 7 (10.6%) |  |  |
| Overweight or obese (≥ 24) | 30 (21.9%) | 3 (7.7%) | | 17 (25.8%) |  |  |
| **History of smoking** |  |  | |  |  |  |
| Never | 50 (36.5%) | 16 (41.0%) | | 25 (37.9%) | χ2 = 1.483, *p =* 0.830 | |
| Previous smoker | 70 (51.1%) | 16 (41.0%) | | 32 (48.5%) |  |  |
| Current smoker | 17 (12.4%) | 7 (18.0%) | | 9 (13.6%) |  |  |
| **History of drinking** |  |  | |  |  |  |
| Never | 54 (39.4%) | 14 (35.9%) | | 33 (50.0%) | χ2 = 4.077, *p =* 0.396 | |
| Previous drinker | 71 (51.8%) | 20 (51.3%) | | 30 (45.5%) |  |  |
| Current drinker | 12 (8.8%) | 5 (12.8%) | | 3 (4.5%) |  |  |
| **Comorbidities (e.g., diabetes, hypertension, stroke)** | | | | | | |
| No | 92 (67.2%) | 19 (48.7%) | | 40 (60.6%) | χ2 = 4.521, *p =* 0.104 | |
| Yes | 45 (32.8%) | 20 (51.3%) | | 26 (39.4%) |  |  |
| **Concurrent medications** |  |  | |  |  |  |
| No | 102 (74.5%) | 20 (51.3%) | | 41 (62.1%) | **χ2 = 8.5434,**  ***p =* 0.014** | |
| Yes | 35 (25.5%) | 19 (48.7%) | | 25 (37.9%) |  |  |
| **History of surgery** |  |  | |  |  |  |
| No | 80 (58.4%) | 23 (59.0%) | | 41 (62.1%) | χ2 = 0.262, *p =* 0.877 | |
| Yes | 57 (41.6%) | 16 (41.0%) | | 25 (37.9%) |  |  |
| **Biomarkers** | | | | | | |
| **Serum** **calcium, mmol/L** |  |  | |  |  |  |
| Normal (≥ 2.20) | 79 (57.7%) | 12 (30.8%) | | 30 (45.5%) | **χ2 = 9.533 *p =* 0.009** | |
| Low (< 2.20) | 58 (42.3%) | 27 (69.2%) | | 36 (54.5%) |  |  |
| **Haemoglobin, g/L** |  |  | |  |  |  |
| Normal (male ≥ 130, female ≥ 120) | 95 (69.3%) | 26 (66.7%) | | 33 (50%) | **χ2 = 7.386, *p =* 0.025** | |
| Low (male ＜ 130, female ＜ 120) | 42 (30.7%) | 13 (33.3%) | | 33 (50%) |  |  |
| **Serum albumin, g/L** |  |  | |  |  |  |
| Normal (≥ 35) | 87 (63.5%) | 7 (17.9%) | | 24 (36.4%) | **χ2 = 30.800,**  ***p <* 0.001** | |
| Low (< 35) | 50 (36.5%) | 32 (82.1%) | | 42 (63.6%) |  |  |
| **White blood cell count, WBC, 10^9^/L** |  |  | |  |  |  |
| Normal (3.5 ~ 9.5) | 125 (91.2%) | 31 (79.5%) | | 60 (90.9%) | χ2 = 4.632 *p =*0*.*099 | |
| Low (< 3.5) or high (> 9.5) | 12 (8.8%) | 8 (20.5%) | | 6 (9.1%) |  |  |
| **Retinol binding protein, RBP, mg/L** |  |  | |  |  |  |
| Normal (25.0 ~ 70.0) | 121 (88.3%) | 25 (64.1%) | | 52 (78.8%) | **χ2 = 12.530,**  ***p =* 0.002** | |
| Low (< 25.0) | 16 (11.7%) | 14 (35.9%) | | 14 (21.2%) |  |  |
| **Serum creatinine, μmol/L** |  |  | |  |  |  |
| Normal (44 ~ 133) | 133 (97.1%) | 38 (97.4%) | | 64 (97.0%) | χ2 = 0.020, *p =* 0.990 | |
| Low (< 44) or high (> 133) | 4 (2.9%) | 1 (2.6%) | | 2 (3.0%) |  |  |
| **Prognostic nutritional index, PNI** | 48.95 (4.93) | 45.25 (5.95) | | 44.75 (6.19) | **H=31.711^a^, *p <* 0.001** | |
| **Neutrophil-to-lymphocyte ratio, NLR** | 1.93 (1.54) | 2.05 (1.28) | | 1.90 (1.23) | H=2.837^a^, *p =* 0.242 | |
| **Platelet-to-lymphocyte ratio, PLR** | 115.70 (52.62) | 149.28 (191.71) | | 119.23 (86.82) | **H=7.412^a^, *p =* 0.025** | |
| **Albumin-to-globulin ratio, AGR** | 1.52 (0.28) | 1.42 (0.22) | | 1.45 (0.31) | H=5.611^a^, *p =* 0.060 | |
| **Modified Glasgow Prognostic Score, mGPS** | | | | | | |
| 0 (normal C-reactive protein and albumin) | 126 (92.0%) | 28 (71.8%) | | 53 (80.3%) | **χ2 = 11.999,**  ***p =* 0.002** | |
| 1, 2 (increased C-reactive protein level) | 11 (8.0%) | 11 (28.2%) | | 13 (19.7%) |  |  |
| **Controlling Nutritional Status Score, CONUT** | | | | | | |
| Normal | 86 (62.8%) | 14 (35.9%) | | 25 (37.9%) | **χ2 = 15.676,**  ***p <* 0.001** | |
| Mild, moderate, or severe malnutrition | 51 (37.2%) | 25 (64.1%) | | 41 (62.1%) |  |  |
| **Carcinoembryonic antigen, CEA, ng/mL** | | | | | | |
| Normal (< 4.7) | 100 (73.0%) | 30 (76.9%) | | 49 (74.2%) | χ2 = 0.247, *p =* 0.884 | |
| High (≥ 4.7) | 37 (27.0%) | 9 (23.1%) | | 17 (25.8%) |  |  |
| **Cancer antigen 199, CA199, U/mL** |  |  | |  |  |  |
| Normal (< 39) | 127 (92.7%) | 31 (79.5%) | | 58 (87.9%) | χ2 = 5.707, *p =* 0.058 | |
| High (≥ 39) | 10 (7.3%) | 8 (20.5%) | | 8 (12.1%) |  |  |
| **NRS2002 score** |  |  | |  |  |  |
| < 3 (no risk of malnutrition) | 124 (90.6%) | 23 (59.0%) | | 57 (86.4%) | **χ2 = 23.102,**  ***p <* 0.001** | |
| ≥ 3 (at risk of malnutrition) | 13 (9.4%) | 16 (41.0%) | | 9 (13.6%) |  |  |
| **Preoperative SMI** |  |  | |  |  | |
| **Preoperative sarcopenia** |  |  | |  |  |  |
| No | 131 (95.6%) | 8 (20.5%) | | 59 (89.4%) | **χ2 = 118.622,**  ***p < .*001** | |
| Yes | 6 (4.4%) | 31 (79.5%) | | 7 (10.6%) |  |  |
| **BMI, kg/m^2^** |  |  | |  |  |  |
| Normal (18.5 ~ < 24.0) | 107 (78.1%) | 23 (59.0%) | | 42 (63.6%) | **H *=* 8.328^a^, *p =* 0.016** | |
| Under weight (< 18.5) | 0 | 13 (33.3%) | | 7 (10.6%) |  |  |
| Overweight or obese (≥ 24) | 30 (21.9%) | 3 (7.7%) | | 17 (25.8%) |  |  |
|  |  |  | |  |  | |

Notes: The scoring methods of CONUT and NRS2002 were referred to: Ignacio de Ulíbarri, J., et al., *CONUT: a tool for controlling nutritional status. First validation in a hospital population.* Nutricion Hospitalaria, 2005. **20**(1): p. 38-45, and Kondrup, J., et al., *Nutritional risk screening (NRS 2002): a new method based on an analysis of controlled clinical trials.* Clinical Nutrition (Edinburgh, Scotland), 2003. **22**(3): p. 321-336, respectively.

**Supplementary S4 Mean scores of each scale of EORTC QLQ-C30 by participant subgroups at different time point**

| **Scale** | **Mild-SML**  **(*n =* 137)** | **Moderate-SML**  **(*n =* 39)** | **Severe-SML**  **(*n =* 66)** | **Difference between subgroups** |
| --- | --- | --- | --- | --- |
| **Mean ± SD at T0 (admission/the day before surgery)** | | | | |
| **Global health status** | 84.55 ± 7.18 | 80.68 ± 5.88 | 81.84 ± 5.69 | **F = 8.376, *p <* 0.001** |
| **Functional scales** |  |  |  |  |
| Physical functioning | 92.26 ± 5.31 | 90.00 ± 9.30 | 88.72 ± 7.52 | **F = 5.003, *p =* 0.007** |
| Role functioning | 95.01 ± 8.42 | 94.19 ± 9.01 | 94.44 ± 8.83 | F = 0.220, *p =* 0.803 |
| Emotional functioning | 94.40 ± 7.43 | 94.32 ± 6.40 | 92.09 ± 7.14 | F = 1.685, *p =* 0.1889 |
| Cognitive functioning | 99.64 ± 2.45 | 99.98 ± 4.01 | 99.57 ± 29.69 | F = 1.080, *p =* 0.341 |
| Social functioning | 93.31 ± 11.15 | 91.66 ± 10.99 | 94.01 ± 6.48 | F = 1.506, *p =* 0.224 |
| **Symptom scales** |  |  |  |  |
| Fatigue | 3.97 ± 7.94 | 4.71 ± 7.04 | 6.27 ± 9.47 | F = 1.275, *p =* 0.281 |
| Nausea and vomiting | 2.68 ± 7.34 | 2.78 ± 6.26 | 3.42 ± 6.82 | F = 0.337, *p =* 0.714 |
| Pain | 1.10 ± 5.03 | 2.27 ± 5.76 | 1.71 ± 5.12 | F = 1.151, *p =* 0.318 |
| Dyspnoea | 4.38 ± 11.30 | 3.03 ± 9.66 | 4.27 ± 11.29 | F = 0.358, *p =* 0.699 |
| Insomnia | 6.57 ± 13.71 | 6.06 ± 12.95 | 8.55 ± 14.74 | F = 0.447, *p =* 0.640 |
| Appetite loss | 10.95 ± 17.67 | 13.64 ± 16.51 | 18.80 ± 16.74 | **F = 3.231, *p =* 0.041** |
| Constipation | 6.08 ± 12.92 | 10.10 ± 15.43 | 8.55 ± 14.74 | F = 1.963, *p =* 0.143 |
| Diarrhea | 4.38 ± 12.00 | 4.04 ± 10.96 | 5.13 ± 12.18 | F = 0.106, *p =* 0.900 |
| Financial difficulties | 0.97 ± 5.63 | 1.52 ± 6.70 | 0.85 ± 5.34 | F = 0.222 *p =* 0.801 |
| **Mean ± SD at T1 (7 days after surgery/discharge)** | | | | |
| **Global health status** | 76.58 ± 9.24 | 71.85 ± 10.31 | 65.38 ± 10.03 | **F = 21.670, *p <* 0.001** |
| **Functional scales** |  |  |  |  |
| Physical functioning | 82.82 ± 11.51 | 74.85 ± 11.20 | 73.33 ± 11.24 | **F = 16.925, *p <* 0.001** |
| Role functioning | 90.88 ± 12.94 | 81.82 ± 13.31 | 85.90 ± 14.07 | **F = 10.837, *p <* 0.001** |
| Emotional functioning | 92.58 ± 8.00 | 86.36 ± 8.73 | 82.05 ± 10.39 | **F = 27.514, *p <* 0.001** |
| Cognitive functioning | 95.86 ± 7.77 | 95.96 ± 7.20 | 93.59 ± 11.22 | F = 1.055, *p =* 0.350 |
| Social functioning | 84.91 ± 13.08 | 82.07 ± 12.85 | 7790.78 ± 12.85 | **F = 4.813, *p =* 0.009** |
| **Symptom scales** |  |  |  |  |
| Fatigue | 19.14 ± 14.37 | 25.59 ± 12.47 | 29.91 ± 12.00 | **F = 11.811, *p <* 0.001** |
| Nausea and vomiting | 6.21 ± 10.50 | 4.04 ± 7.77 | 8.12 ± 10.73 | F = 2.437, *p =* 0.090 |
| Pain | 16.30 ± 16.48 | 11.62 ± 14.01 | 19.66 ± 17.47 | **F = 3.426, *p =* 0.034** |
| Dyspnoea | 9.49 ± 15.63 | 8.59 ± 14.69 | 11.11 ± 17.66 | F = 0.316, *p =* 0.729 |
| Insomnia | 19.22 ± 19.27 | 22.22 ± 17.86 | 18.80 ± 22.68 | F = 0.611, *p =* 0.543 |
| Appetite loss | 24.57 ± 27.20 | 28.79 ± 24.73 | 35.04 ± 22.88 | F = 2.609, *p =* 0.076 |
| Constipation | 7.79 ± 14.15 | 5.05 ± 12.04 | 6.84 ± 13.63 | F = 0.911, *p =* 0.404 |
| Diarrhea | 3.89 ± 11.48 | 5.56 ± 13.82 | 4.27 ± 11.29 | F = 0.421, *p =* 0.657 |
| Financial difficulties | 10.46 ± 20.11 | 15.15 ± 17.72 | 24.78 ± 19.82 | **F = 8.384, *p < .*001** |
| **Mean ± SD at T2 (1 month after surgery)** | | | | |
| **Global health status** | 71.90 ± 10.11 | 68.57 ± 10.07 | 64.10 ±10.50 | **F = 9.019, *p <* 0.001** |
| **Functional scales** |  |  |  |  |
| Physical functioning | 75.77 ± 12.12 | 69.39 ± 10.9180 | 71.28 ± 11.46 | **F = 7.304, *p =* 0.001** |
| Role functioning | 86.37 ± 13.59 | 85.35 ± 12.58 | 83.76 ± 13.51 | F = 0.612, *p =* 0.543 |
| Emotional functioning | 89.54 ± 9.48 | 84.22 ± 8.54 | 81.41 ± 9.65 | **F = 15.154, *p <* 0.001** |
| Cognitive functioning | 94.04 ± 8.75 | 93.69 ± 9.14 | 93.59 ± 11.22 | F = 0.029, *p =* 0.972 |
| Social functioning | 90.51 ± 12.09 | 86.62 ± 12.48 | 82.48 ± 12.06 | **F = 7.310, *p =* 0001** |
| **Symptom scales** |  |  |  |  |
| Fatigue | 17.35 ± 13.33 | 23.90 ± 12.96 | 27.91 ± 11.36 | **F = 12.664, *p <* 0.001** |
| gNausea and vomiting | 6.94 ± 10.82 | 7.83 ± 10.22 | 6.41 ± 9.06 | F = 0.510, *p =* 0.601 |
| Pain | 12.53 ± 16.64 | 10.86 ± 11.13 | 15.81 ± 13.76 | F = 1.362, *p =* 0.258 |
| Dyspnoea | 6.57 ± 13.31 | 5.56 ± 12.52 | 5.98 ± 12.96 | F = 0.141, *p =* 0.869 |
| Insomnia | 15.08 ± 18.51 | 17.17 ± 18.71 | 18.80 ± 22.68 | F = 0.741, *p =* 0.418 |
| Appetite loss | 19.71 ± 23.76 | 26.18 ± 24.84 | 33.19 ± 26.57 | **F = 5.073, *p =* 0.007** |
| Constipation | 4.38 ± 11.30 | 7.07 ± 13.73 | 7.69 ± 14.22 | F = 1.655, *p =* 0.193 |
| Diarrhea | 5.84 ± 13.34 | 5.05 ± 13.39 | 2.56 ± 9.00 | F = 1.000, *p =* 0.369 |
| Financial difficulties | 6.81 ± 14.65 | 9.60 ± 16.29 | 7.69 ± 16.15 | F = 0.732, *p =* 0.482 |
| **Mean ± SD at T3 (3 months after surgery)** | | | | |
| **Global health status** | 81.45 ± 11.34 | 72.73 ± 10.80 | 69.66 ± 13.31 | **F = 22.581, *p <* 0.001** |
| **Functional scales** |  |  |  |  |
| Physical functioning | 90.12 ± 10.29 | 81.52 ± 10.64 | 80.18 ± 12.09 | **F = 21.751, *p <* 0.001** |
| Role functioning | 92.94 ± 12.58 | 87.63 ± 13.50 | 86.75 ± 13.88 | **F = 5.617, *p =* 0.004** |
| Emotional functioning | 93.55 ± 8.57 | 85.35 ± 8.90 | 83.76 ± 10.11 | **F = 29.239, *p <* 0.0018** |
| Cognitive functioning | 94.16 ± 8.95 | 92.93 ± 9.72 | 94.87 ± 7.79 | F = 0.667, *p =* 0.514 |
| Social functioning | 92.82 ± 12.42 | 89.39 ± 12.28 | 84.61 ± 12.48 | **F = 7.092, *p =* 0.001** |
| **Symptom scales** |  |  |  |  |
| Fatigue | 12.08 ± 11.94 | 19.36 ± 12.68 | 23.93 ± 13.38 | **F = 17.291, *p <* 0.001** |
| Nausea and vomiting | 6.33 ± 9.72 | 10.36 ± 9.13 | 11.54 ± 9.49 | **F = 7.923, *p <* 0.001** |
| Pain | 10.71 ± 15.75 | 8.59 ± 11.78 | 8.55 ± 12.01 | F = 0.670, *p =* 0.512 |
| Dyspnoea | 5.60 ± 12.50 | 5.56 ± 12.52 | 4.27 ± 12.29 | F = 0.185, *p =* 0.831 |
| Insomnia | 8.76 ± 14.72 | 13.64 ± 18.47 | 16.24 ± 16.88 | **F = 4.200, *p =* 0.016** |
| Appetite loss | 18.00 ± 22.51 | 24.24 ± 21.52 | 23.93 ± 21.56 | F = 2.284, *p =* 0.104 |
| Constipation | 5.11 ± 12.05 | 4.55 ± 11.53 | 5.98 ± 12.96 | F = 0.174, *p =* 0.840 |
| Diarrhea | 6.33 ± 13.73 | 5.05 ± 12.04 | 7.69 ± 14.23 | F = 0.493 *p =* 0.612 |
| Financial difficulties | 2.19 ± 8.29 | 3.03 ± 9.66 | 4.27 ± 11.29 | F = 0.819, *p =* 0.442 |

**Supplementary S5 Correlations between potential predictors of moderate/severe muscle loss**


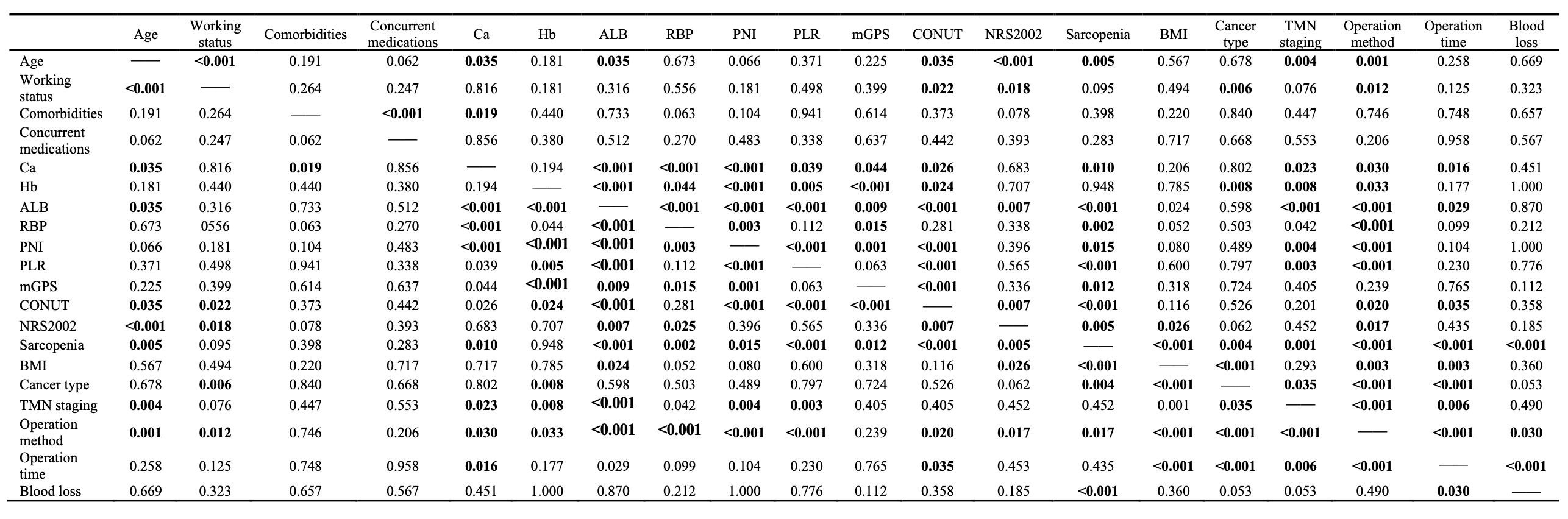


Notes: Ca = Serum calcium; Hb = Haemoglobin; ALB = Serum albumin; RBP = Retinol binding protein; PNI = Prognostic nutritional index; PLR = Platelet-to-lymphocyte ratio; mGPS = Modified Glasgow Prognostic Score; CONUT = Controlling Nutritional Status Score; NRS = Nutritional risk screening.

**Supplementary S6 Univariate logistic regression for predictors of moderate/severe muscle loss**

| **Variables** | **OR (95% CI)** | ***P*** |
| --- | --- | --- |
| **Age, years** |  |  |
| < 65 | 1.00 |  |
| ≥ 65 | 4.560（2.627-7.914） | **< 0.001** |
| **Comorbidities** |  |  |
| No | 1.00 |  |
| Yes | 1.197 (0.709-2.022) | 0.501 |
| **NRS2002 score** |  |  |
| < 3 | 1.00 |  |
| ≥ 3 | 2.981 (1.441-6.165) | **0.003** |
| **Preoperative sarcopenia** |  |  |
| No | 1.00 |  |
| Yes | 14.973 (5.633-39.802) | **< 0.001** |
| **Serum calcium, mmol/L** |  |  |
| Normal | 1.00 |  |
| Low, < 2.20 | 2.105 (1.255-3.532) | **0.005** |
| **Haemoglobin, g/L** |  |  |
| Normal | 1.00 |  |
| Low, (male＜130, female＜120) | 1.704 (1.005-2.890) | **0.048** |
| **Retinol binding protein, RBP, mg/L** |  |  |
| 25.0 ~ 70.0 | 1.00 |  |
| < 25.0 | 3.514 (1.752-7.051) | **< 0.001** |
| **Prognostic nutritional index, PNI** |  |  |
| > 45 | 1.00 |  |
| ≤45 | 7.851 (4.194-14.696) | **< 0.001** |
| **Platelet-to-lymphocyte ratio, PLR** |  |  |
| ≤160 | 1.00 |  |
| > 160 | 3.597 (1.902-6.803) | **< 0.001** |
| **Modified Glasgow Prognostic Score, mGPS** | | |
| 0 | 1.00 |  |
| 1,2 | 3.394 (1.577-7.303) | **0.002** |
| **Controlling Nutritional Status Score, CONUT** | | |
| Normal | 1.00 |  |
| Mild, moderate or severe malnutrition | 2.945 (1.738-4.998) | **< 0.001** |
| **Cancer type** |  |  |
| Colorectal cancer | 1.00 |  |
| Esophageal cancer | 2.745 (1.148-6.565) | **0.023** |
| Gastric cancer | 1.269 (0.772-2.232) | 0.407 |
| **TNM staging** |  |  |
| I, II | 1.00 |  |
| Ⅲ, IV | 6.439 (3.655-11.345) | **< 0.001** |
| **Operation method** |  |  |
| Open surgery | 1.00 |  |
| Laparoscopic or robotic surgery | 0.156 (0.085-0.285) | **< 0.001** |
